# Processing serial crystallography data with *CrystFEL*: a step-by-step guide

**DOI:** 10.1107/S205979831801238X

**Published:** 2019-01-31

**Authors:** Thomas A. White

**Affiliations:** a Center for Free-Electron Laser Science, Deutsches Elektronen-Synchrotron DESY, Notkestrasse 85, Hamburg, Germany

**Keywords:** *CrystFEL*, serial crystallography, X-ray free-electron lasers, data processing

## Abstract

This article provides a step-by-step guide to the use of the *CrystFEL* software for processing serial crystallography data from an X-ray free-electron laser or a synchrotron light source. Whereas previous papers have described the theory and algorithms and their rationale, this paper describes the steps to be performed from a user perspective, including command-line examples.

## Abstract 2

A step-by-step guide to processing serial crystallography data from X-ray free-electron lasers and synchrotron sources using *CrystFEL* is provided.

## 1. Introduction   

Serial crystallography (SX) techniques, in which a single diffraction snapshot is recorded from each of a large number of crystals, have recently become popular for use at X-ray free-electron laser (XFEL) facilities (Chapman *et al.*, 2011 ▸) and synchrotron light sources (Stellato *et al.*, 2014 ▸). They represent part of a significant recent trend towards large data sets in crystallography, requiring automated data-processing pipelines and large-scale computing environments. *CrystFEL* was created to meet the needs arising from this trend, as a piece of software for processing serial crystallography data sets consisting of large numbers of essentially unrelated diffraction snapshots.

*CrystFEL* was first released in 2012 (White *et al.*, 2012 ▸). It is free and open-source software (Ince *et al.*, 2012 ▸), which means that the full source code is provided, with freedom to study the code and make changes if necessary. Since the first version, incremental improvements to *CrystFEL* have had a significant impact on the scientific outcomes of experiments by improving the quality of the information that it can extract from a given set of raw data (Nass *et al.*, 2016 ▸)

*CrystFEL* can be downloaded from the *CrystFEL* website at https://www.desy.de/~twhite/crystfel. The website also contains a large amount of other information, including a tutorial, installation instructions, best-practice guidelines, frequently asked questions, changes between versions, presentation slides, a list of citations and programming-interface information for developers.

**Figure 1:**
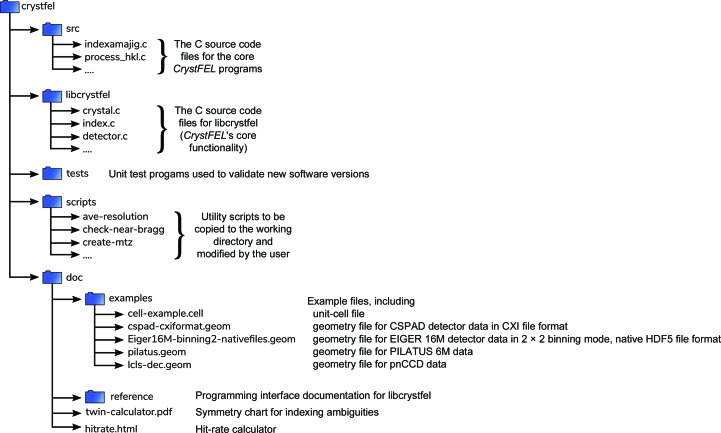
Layout of the *CrystFEL* download archive.

Fig. 1 ▸ shows the layout of the most important folders in the *CrystFEL* package. *CrystFEL* is a suite of software comprising 15 core programs: *indexamajig*, *ambigator*, *process_hkl*, *partialator*, *compare_hkl*, *check_hkl*, *cell_explorer*, *geoptimiser*, *hdfsee*, *list_events*, *render_hkl*, *whirligig*, *get_hkl*, *partial_sim* and *pattern_sim*. *CrystFEL* is primarily a command-line-driven piece of software, with some exceptions which will be detailed later. Once installed, reference documentation for these programs can be obtained using the standard Unix manual system by typing man indexamajig (or any other program name) at the command line. A top-level manual page, accessed via man crystfel, gives an overall introduction. The manual pages are also available on the *CrystFEL* website.

**Figure 2:**
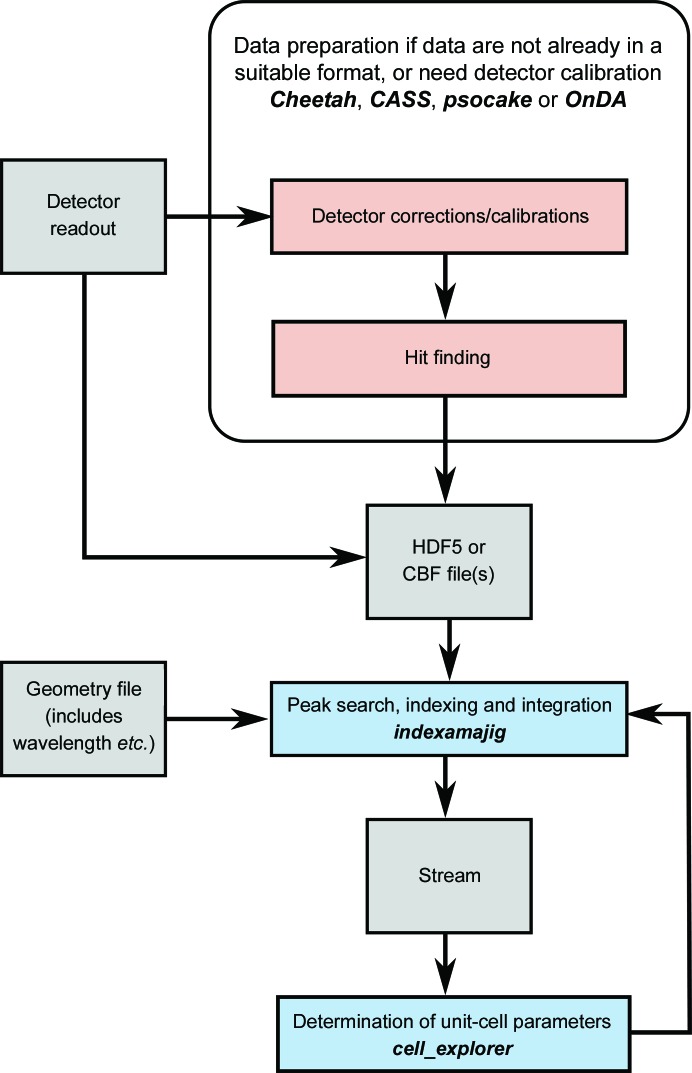
Flow diagram for data import, indexing and integration with *CrystFEL*. Blue and red boxes indicate programs internal and external to *CrystFEL*, respectively, with program names in bold italic text. Grey boxes indicate files or data.

The overall flow of data processing is shown in Figs. 2 ▸, 3 ▸, 4 ▸ and 5 ▸, including the programs which are needed at each stage. The diagrams are not exhaustive, but show the main pathway through processing a data set, starting with preparing the data and ending with importing the data into external programs for structure solution.

This article describes the processing pipeline using *CrystFEL* v.0.7.0, which is the latest version at the time of writing. Basic knowledge of the Unix command-line environment will be assumed. Lines beginning with $ indicate examples of commands, in which line breaks should be ignored, *i.e.* they should be entered on a single line.

In addition to the core programs, the *CrystFEL* package contains a repository of scripts which are intended to be copied to the working directory and customised to suit the individual situation. To make these scripts readily accessible, it is helpful to download a separate copy of *CrystFEL* even if it has been installed centrally on a facility computer system. After copying a script to the working directory, it will usually be necessary to mark it as executable using chmod +x.

While this article attempts to describe alternative possibilities at each processing step, the supporting information contains a complete worked example for some freely available data. In the worked example, the full sequence of commands can be seen with only short comments, but including the expected outputs from each program.

## 2. Preparing the data   

As with almost any computational data-processing procedure, the first task is to put the data into a file format that can be read by the software. *CrystFEL* can read image data in Crystallographic Binary Format (CBF) or Hierarchical Data Format v.5 (HDF5). HDF5 is itself a ‘container format’, meaning that the data inside it can be in variety of layouts. For example, one file on disk might contain a single two-dimensional image, or many two-dimensional images stacked together to form a three-dimensional array. The range of HDF5 layouts usable by *CrystFEL* has been described previously (White, Mariani *et al.*, 2016 ▸) and includes the formats written by *Cheetah* (Barty *et al.*, 2014 ▸), *CASS* (Foucar, 2016 ▸), *OnDA* (Mariani *et al.*, 2016 ▸) and *psocake* (Shin *et al.*, 2018 ▸), which all use their own layouts. The NeXus standard (Bernstein *et al.*, 2014 ▸; Könnecke *et al.*, 2015 ▸) is based on HDF5 and can also be used by *CrystFEL*. Dectris EIGER detectors write data in NeXus HDF5 format.

Duplication of the data in a new format should be avoided wherever possible, particularly if the total size of the data is very large. The support of *CrystFEL* for CBF and EIGER formats means that no data-format conversion step will be necessary for many synchrotron experiments. For other experiments, notably those performed using the Linac Coherent Light Source (LCLS), the data will need to be converted from the specialised format written by the data-acquisition system. In the case of LCLS, this format is known as eXtended Tagged Container (XTC; Thayer *et al.*, 2016 ▸). At the same time as converting the format, detector-calibration steps such as subtracting background signals may need to be performed. If a format conversion or detector-readout intensity calibration step is necessary, it is best combined with a ‘hit-finding’ step to reduce the amount of data duplicated. This means that output files are written only for frames which appear to contain Bragg spots and therefore have a good chance of being useful.

The initial stages of file-format conversion, detector-readout calibration and hit finding, are beyond the scope of *CrystFEL* and therefore also beyond the scope of this article. Unfortunately, owing to the many differences between facility data formats and processing environments, this can be one of the most challenging steps. Several programs are available, including *Cheetah*, *CASS*, *OnDA* and *psocake*, which have all been mentioned above. Tutorials are available on the web for *Cheetah* (https://www.desy.de/~barty/cheetah/Cheetah/), including the steps necessary to get started at various facilities, and *psocake* (https://confluence.slac.stanford.edu/display/PSDM/Psocake+SFX+tutorial). For SX experiments at the SPring-8 Ångstrom Compact Laser (SACLA), a data-processing pipeline is provided which writes the hits in HDF5 format using *Cheetah* (Nakane *et al.*, 2016 ▸), and a tutorial is also available (https://github.com/biochem-fan/cheetah/wiki).

*CrystFEL* requires a ‘detector geometry file’, the purpose of which is twofold. Firstly, it tells *CrystFEL* how the data are laid out in the input file, for instance whether there is just one frame or many frames per file. Secondly, it tells *CrystFEL* how the detector is laid out in physical space. Complicated multi-panel detectors are common in XFEL experiments (Philipp *et al.*, 2010 ▸; Kameshima *et al.*, 2014 ▸; Allahgoli *et al.*, 2015 ▸), and the geometry file describes the position and orientation of each panel in three dimensions, as well as additional constants such as the pixel size and the photon energy. The photon energy can vary from frame to frame because of the operating principle of an XFEL (Bonifacio *et al.*, 1994 ▸), and so a location within the data file containing the energy for each frame can be given instead of a fixed number for all of the frames. The geometry file hence contains all of the information that is needed to interpret the contents of the data file as the physical setup of a diffraction experiment. As with almost all files used by *CrystFEL*, the geometry file is a plain-text file, and documention of the format can be accessed with the command man crystfel_geometry.

Unfortunately, like hit finding and detector-readout intensity calibration, creating and refining a geometry file for a complicated multipanel detector is a daunting task, and is itself the subject of entire papers (Yefanov *et al.*, 2015 ▸; Ginn & Stuart, 2017 ▸; Brewster *et al.*, 2014 ▸). Some example geometry files are distributed with *CrystFEL*, which can be used as templates, including examples for the CSPAD detector at LCLS in two different datafile layouts (see Fig. 1 ▸). The LCLS publishes a repository of geometry files for its detectors on its website (https://confluence.slac.stanford.edu/display/PSDM/Geometry+History). Geometrical information about the detector and its position relative to the beam should be available from the X-ray facility as a rough starting point. As a general guideline, to get started with processing a data set, the panel positions need to be accurate to much less than the smallest separation between Bragg peaks which will be seen in any pattern. Once a few patterns can be processed, the geometry can be refined as described in Section 3.4;3.4. For the final data analysis, the geometry should reach subpixel accuracy.

The final preparatory task is to create a list of the files to process in text format. This can easily be performed using standard Unix command-line tools, for example, to create files.lst containing a list of all CBF files in the folder data:
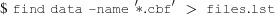
If the data are stored in files which contain more than one frame each, the procedure is the same. *CrystFEL* will recognise that each file contains more than one frame and process every frame individually. If all of the data are contained in a single file, then there need only be one filename in the list. The list of input files need not include every frame of data, allowing selective processing of data. To perform this when each file contains multiple frames, the program *list_events* can expand the list of multiframe files to a full list of frame identifiers, which can then be altered as required.

## 3. Indexing and integration   

The flow of data preparation, indexing and integration is shown in Fig. 2 ▸. The heart of *CrystFEL* is the indexing and integration tool *indexamajig*. This program reads the list of filenames or event descriptors prepared earlier, reads the image data and executes the indexing and integration pipeline. A flowchart for the pipeline has been given previously (White, Mariani *et al.*, 2016 ▸). The first step is to find the locations of the obvious Bragg peaks in the image. These are then used to index the pattern, which is followed by a series of refinement and result-checking steps. If the indexing solution is accepted, the unit-cell parameters and orientation, combined with the detector geometry, wavelength and other information about the X-ray beam, are used to calculate the positions of the Bragg peaks in the frame. Their intensities are then measured from the image data. In this way, the intensities are measured not only for the strong reflections but also for the weak or absent reflections, which are equally important for solving the structure. The output from *indexamajig* is known as a stream file. It is a long text file which contains the indexing and integration results, as well as other information such as the locations of peaks and the unit-cell parameters, for each frame in succession.

To begin processing the data, the list of files, geometry file and output stream filename need to be given to *indexamajig*. For example, for a list of files called files.lst, a geometry file called my.geom and an output filename my.stream, the command line would be
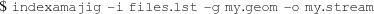
The command-line option -i specifies the input file, -g the geometry file and -o the output stream. In practice, additional parameters will be needed to tune the algorithms, as described below.

### 3.1. Setting up peak detection   

Accurate peak detection is important for successful indexing. If too many spots are missed there may not be sufficient information for the indexing algorithm and subsequent refinement algorithms. If too many spurious spots are included, the indexing may not be able to find the true lattice repeats in the pattern. It therefore pays to take some time to optimise the peak-detection parameters. *CrystFEL* offers a choice of peak-detection algorithms. The simpler algorithms have fewer parameters to tune but are more susceptible to noise.

If the raw data have been processed using *Cheetah*, the data files will usually contain lists of peak positions. Hit finding in *Cheetah* is performed by finding peaks in each pattern and accepting frames which contain at least a certain minimum number of peaks. Since the peak detection needs to be tuned in *Cheetah* anyway, it makes sense to reuse the peak-detection results in *CrystFEL* rather than to perform new peak detection. This can be performed by adding the following option to the *indexamajig* command line if *Cheetah* has been configured to generate single-frame data files,
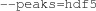
or the following if *Cheetah* has been configured to generate multiframe data files in CXI format,
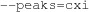



For other types of input data, the peak-detection algorithms built into *CrystFEL* should be used. The simplest of these is a gradient-based search (Zaefferer, 2000 ▸),
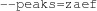
This algorithm has three tunable parameters: the threshold, the minimum gradient and the minimum signal-to-noise ratio. Peak detection is triggered when the pixel value is above the threshold and the local gradient of the pixel values exceeds the minimum gradient. For the candidate peak to be accepted, its total intensity must be greater than the specified number of times its estimated error. These three parameters are given using the following three options for *indexamajig*; for example for a threshold of 100 detector units (adu), a minimum gradient of 100 adu per pixel and a minimum signal-to-noise ratio of 5,
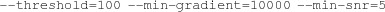
Owing to a historical mistake, the parameter given to --min-gradient is actually the *square* of the required gradient measured in adu per pixel. This has not been changed because of the importance of maintaining compatibility with earlier versions and giving consistent results with the same input parameters.

The appropriate values for the peak-detection parameters depend strongly on the type of detector, the strength of the Bragg peaks and the amount of background scattering. Initial values can be determined by examining the image data. For this, any image viewer can be used. *CrystFEL* offers a simple viewer called *hdfsee*, which can be invoked as follows, in this case for an image file called image.cbf and a geometry file called my.geom,
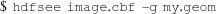
For simple data layouts, the geometry-file argument (-g my.geom) can be omitted. However, using the geometry file allows *hdfsee* to show the image data in the correct physical layout. Once started, the pixel values can be examined by opening the ‘View Numbers’ window from the ‘Tools’ menu. Clicking anywhere on the image results in the pixel values in that vicinity being shown in this window. Using this, the background level and heights of typical peaks can be estimated. The initial threshold should be a slightly larger value than most of the background pixels, but much smaller than the Bragg peaks. The initial (squared) gradient should be the square of about half of the difference between the average background and typical Bragg peak intensities. A value of 3 is usually a suitable initial estimate for the signal-to-noise ratio, provided that the geometry file contains the correct value for the number of detector intensity units per photon.

Another peak-search algorithm in *CrystFEL* is called *peakfinder*8. This algorithm originated in *Cheetah* (Barty *et al.*, 2014 ▸) and was incorporated in *CrystFEL* because it gives better results when the background intensity varies radially but has approximate circular symmetry. This algorithm searches for peaks above a radius-dependent threshold intensity, checks their signal-to-noise ratio and also requires that peaks contain a certain minimum number of pixels above the threshold. The command-line options for this peak-search algorithm can be found on the *indexamajig* manual page, which is accessed using the command man indexamajig.

To test the peak-detection parameters, *indexamajig* should be run on the first few frames of the data set. Although not necessary, it simplifies matters to also tell *indexamajig* not to proceed with indexing and integrating each pattern at this stage. This can easily be performed by adding --indexing=none, an option which will be described in more detail in the next section. The full command line, combining the elements described above, might look like
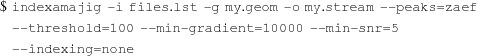



*indexamajig* will periodically display progress updates, including the number of frames processed. After sufficient frames have been processed for inspection (about 100), it can be interrupted by pressing Ctrl+C, the normal command-line interrupt keystroke.

The stream file written by *indexamajig* can then be used to make an initial evaluation of the accuracy of the peak detection. A simple script has been provided for this purpose, called *check-peak-detection*. This script opens *hdfsee* sequentially for each frame, each time displaying the image with spot positions circled. If many false peaks are seen, the values of the peak-finding parameters should usually be increased to make the detection more stringent. If many real peaks are missed, the values should usually be decreased. After checking a frame, closing the *hdfsee* window will cause the script to open it again with the next frame. The script should be copied into the working directory from the scripts folder in the *CrystFEL* download package, marked as executable and then run, as follows:
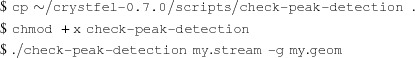
As might be expected, the geometry file should be given to allow the images to be displayed in a physically realistic layout. Here, it is assumed that the *CrystFEL* download package is located in the home directory (∼).

Another option for checking the peak detection is to use the *cxiview* program from the *Cheetah* package. At the cost of installing a separate piece of software, this program offers a more comfortable experience, such as the ability to move backwards, skip frames or jump randomly among the frames in the stream, instead of viewing them in sequence. It also allows the peaks from the peak search and the calculated spot locations to be viewed together. Documentation for *cxiview* is available on the WWW at https://www.desy.de/~barty/cheetah/Cheetah/cxiview.html.

Once the peak finding is approximately satisfactory, it can be further refined with reference to the fraction of indexed patterns, as described in Section 3.3.

There are a few more parameters which will need to be determined at this stage for the best final data quality, namely the size of the integration regions used to measure the intensities of the reflections and the background around them. When integrating a reflection, *CrystFEL* will consider a circular region centred on the calculated reflection position, which should contain the peak itself, and an annulus further out, from which it estimates the background underneath the peak (White *et al.*, 2013 ▸). Three parameters need to be determined: the radius of the peak region and the inner and outer radii of the background annulus. The default parameters (four, five and seven pixels, respectively) are usually appropriate for data with sharp, widely separated Bragg peaks, but for less sharp peaks or closer spot separation they may need to be altered. This can be performed visually while inspecting the peak-detection results using *hdfsee* via the *check-peak-detection* script. Using the ‘View’ menu, the binning of the image can be set to 1, so that it is displayed pixel-for-pixel on the screen. The radii of the circles used to indicate the peak positions can also be set via the ‘View’ menu, allowing them to be visualised. The peak radius should be set such that the entirety of each peak is within the circle, with a pixel or two of buffer region to allow for residual inaccuracy in the detector geometry. The inner and outer background radii should be set such that the annulus falls entirely in the gaps between rows of reflections as far as possible, but otherwise is as large as possible. The three radii (peak, background inner and outer respectively) should then be given to *indexamajig* using the option --int-radius, as follows
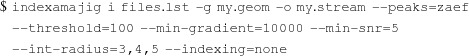



Finally, if the input data contain a large number of empty patterns, it will make the processing faster if patterns with a small number of peaks are skipped over. This can be performed using the option --min-peaks; for example, to ignore all patterns with fewer than 50 peaks this would be --min-peaks=50.

### 3.2. Determining the unit cell   

*CrystFEL* offers a choice of indexing methods. Most of the methods involve calling external indexing programs such as *MOSFLM* (Powell, 1999 ▸), *DirAx* (Duisenberg, 1992 ▸), *FELIX* (Beyerlein *et al.*, 2017 ▸) or *XDS* (Kabsch, 1988 ▸, 2010 ▸). Other methods are built into *CrystFEL*, including an implementation of the *TakeTwo* algorithm (Ginn *et al.*, 2016 ▸). The indexing methods can be selected using the --indexing option in *indexamajig*. For example, the following option would select *MOSFLM*, using *DirAx* as a fallback if indexing with *MOSFLM* is not successful:
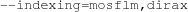
The sequence of fallback indexing methods can be any length, or a single indexing method can be used. Indexing can also be disabled completely by specifying ‘none’ as the indexing method, as was used above to set up peak detection.

To make things easier, *CrystFEL* v.0.7.0 can automatically determine which indexing methods are available. Methods will be added to the list, in a preset order of priority, if the corresponding programs are installed on the computer. Some indexing methods require the unit-cell parameters to be known in advance, and these methods will be automatically added to the list when parameters are provided (see Section 3.3). Automatic selection of the indexing methods will occur unless the --indexing parameter is given to *indexamajig*. Therefore, the only change needed to enable indexing is to remove the --indexing=none option from the previous command line:
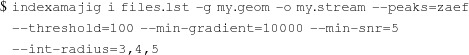



Usually, the more indexing methods that can be used, the higher the overall indexing fraction will be. However, enabling more indexing methods will make the processing take longer. When determining the unit cell, it is usually better to restrict the indexing to one method because different programs may produce different representations of the same unit cell. For example, *DirAx* does not handle centred unit cells and will always give a primitive representation of a centred cell. *MOSFLM* is a good choice because it runs quickly but also offers the ability to use prior lattice-type information if available.

In *CrystFEL* v.0.7.0, the order of priority for automatic determination places the fastest methods first. *MOSFLM* comes first of all, because it runs quickly but also offers many features such as the ability to use prior information. *XDS* is placed last, since in our experience it is the least successful at indexing snapshot patterns. *TakeTwo* and *FELIX* are excluded from automatic determination because they can take a long time to run. The order of indexing methods should not normally need to be overridden.

Indexing frames takes significantly longer than just peak detection. Therefore, at this stage it may be useful to instruct *indexamajig* to process several frames in parallel. This can be performed by simply adding the option -j *n*, where *n* is the number of frames which should be processed at once. Usually, *n* should equal the number of processors in the computer. This type of multiprocessing only works within one computer, whereas Section 6.3 describes how to multiprocess across several computers in a cluster environment.

If no prior information about the unit-cell parameters is given, *indexamajig* will index the patterns freely, meaning that the indexing algorithms will be required to determine the six unit-cell parameters (*a*, *b*, *c*, α, β, γ) and the crystal orientation (three further parameters). If everything goes well, the unit-cell parameters should have similar values for all frames. In practice, there will be some variation owing to experimental error and also some outlying sets of parameters. Since the output from *indexamajig* is a simple text file, albeit a large one, it is quite easy to find the unit-cell parameters. They appear on lines in the stream starting with the text ‘Cell parameters’. To conveniently inspect the overall distributions of parameters, the *CrystFEL* program *cell_explorer* can be used. It is invoked very simply:
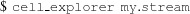



The graphical user interface of *cell_explorer* is shown in Fig. 6 ▸. It reads the unit-cell parameter information for every frame in the stream and plots histograms for the six unit-cell parameters, using colour coding to represent the different centring types. It allows the graphs to be panned (by click/dragging the mouse), zoomed in or out (using the scroll wheel) and the histogram binning to be altered (using the + and − keys). By click/dragging the mouse with the shift key held down, a range of values can be selected for one of the parameters. If this is performed, any set of unit-cell parameters which has that parameter outside the selected range will be excluded. This allows the true parameters to be extracted, even if there is a complicated mixture of different cells (whether this is owing to problems with the data processing or because there is truly a mixture of different lattices among the crystals).

The true unit-cell parameters should appear as strong, sharp peaks in the histograms. Otherwise, a problem with the earlier data processing should be suspected; the most common cause is an inaccurate detector-geometry file, such as an incorrect beam-centre location or an incorrect camera-length value. Once a plausible set of parameters has been found, *cell_explorer* can fit curves to the selected peaks. To perform this, the peaks for all parameters should first be selected (using shift+click/drag) and the fitting procedure then triggered using ‘Fit cell’ in the ‘Tools’ menu or by pressing Ctrl+F. The fitted values will be displayed on each histogram, including an estimated standard deviation, and can be exported (using ‘Create unit cell file’ under the ‘File’ menu) to a text file for the next step.

### 3.3. Indexing and integrating using unit-cell constraints   

Providing expected unit-cell parameters to *indexamajig* enables it to reject indexing solutions which do not match. It is obviously essential for merging the data that the unit-cell parameters for all crystals are consistent. In addition, providing unit-cell parameters allows the use of indexing algorithms which determine only the orientation of the crystal, which is in principle an easier task. The *TakeTwo* (Ginn *et al.*, 2016 ▸) and *FELIX* (Beyerlein *et al.*, 2017 ▸) indexing algorithms belong to this category. Some indexing programs, such as *MOSFLM*, can make use of prior unit-cell information but do not require it, and this is also taken into account by *indexamajig*. Therefore, the only change that needs to be made to perform this step is to provide the unit-cell parameter file written by *cell_explorer*, which is performed using the option -p, 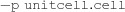



An example of a full command line for *indexamajig*, including the option for multiprocessing on a four-processor machine, is therefore as follows:
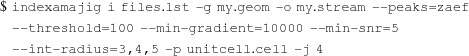



The ‘indexing rate’, which is defined as the fraction of frames in which at least one crystal lattice could be found, will periodically be displayed. It is normal for the indexing rate in this step to be lower than in the previous step, because all spurious indexing solutions which do not match the expected results are now being rejected. The overall indexing rate will be reported just as the *indexamajig* program finishes. The indexing rate is more sensitive than a visual analysis; therefore, it might be possible to increase the indexing rate by altering the peak-detection parameters from the values determined earlier or even trying a different peak-detection method (see Section 3.1). Making the peak detection more stringent may improve the results by avoiding spurious peaks, but making it less stringent may also help by providing more data points to the indexing algorithms. Slightly altering the detector geometry, for example by testing 0.5 or 1 mm either side of the current sample-to-detector distance in the geometry file, may also improve the indexing rate. Therefore, at least a few rounds of indexing should be performed before accepting the result as final.

### 3.4. Checking the quality of indexing   

The internal indexing logic of *CrystFEL* already performs several tests to check that the indexing solution is correct. Despite these checks, it is possible to obtain incorrect indexing solutions, especially if the geometry is not accurate or the peak-search parameters are not set appropriately. Therefore, it is important to check the alignment of peaks visually, just as before when setting up the peak detection. A script called *check-near-bragg* is provided which behaves similarly to *check-peak-detection* (described above) except that it shows the calculated (‘predicted’) reflection locations instead of the peak locations identified by the peak search,
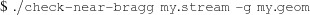
Alternatively, the *cxiview* program from *Cheetah* can also be used. Some experience is needed to be able to judge whether or not the spot positions are correct. The most important thing is that the patterns of reflections agree; for example, a line or arc of reflections should correspond to a line or arc of true spots, even if there might be an excessive number of reflections predicted either side of the true spots. If there are a large number of patterns where the calculated reflection patterns do not agree with the true spots, the earlier stages should be revisited.

The prediction-refinement algorithm also refines the position of the central beam on the detector for each frame. The resulting shifts are stored in the stream, and these should also be checked using a scatter plot. This can be performed using a script called *detector-shift* provided with *CrystFEL*:
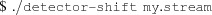



The best outcome is for the shifts to be clustered tightly around the origin of the graph. Successful indexing despite an initially incorrect beam position will show as a cluster of points away from the origin (for an example, see Fig. 4 in White, Mariani *et al.*, 2016 ▸). This script can also be used to correct the offset by running it again, this time telling it the name of the geometry file which should be updated:
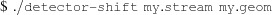
This command will result in my-predrefine.geom (the filename for the input geometry suffixed with -predrefine) being created, which should be used for a new run of indexing to improve the overall results.

The detector-shift tool can only correct offsets of the entire detector in two directions perpendicular to the beam direction (note that the detector panels need not necessarily be in this plane). A more advanced program in *CrystFEL* called *geoptimiser* can be used to refine the detector geometry down to the level of individual panels. The way that *geoptimiser* works has been described in detail in a separate paper (Yefanov *et al.*, 2015 ▸). In particular, it allows the panels to be handled in groups which are either treated rigidly or used to calculate adjustments for panels for which there is not sufficient information for an individual refinement (by using the average of the adjustments for the other panels in the group). A few thousand indexed patterns should be used for a good refinement, and a typical command line for *geoptimser* would be as follows:
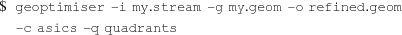
Here, the stream my.stream will be used to refine geometry file my.geom to produce refined.geom, treating panels grouped in asics as strictly rigid with respect to one another and using panels grouped in quadrants for the adjustment of panels with a low number of measurements. The names asics and quadrants are defined in the geometry file. For detectors which can be treated as single panels, which includes most detectors at synchrotron beamlines to a good approximation, it will not be necessary to use these panel-grouping options.

As before, the indexing step should be repeated with the refined geometry file.

## 4. Resolving an indexing ambiguity   

Certain symmetry classes are subject to indexing ambiguities. These occur when the crystal can be rotated, usually by 180°, in such a way that the spot positions stay exactly (or very nearly exactly) the same but the structure is not identical to before. The situations in which this can happen, and algorithms to address the problem, have been amply discussed previously (White *et al.*, 2013 ▸; White, Mariani *et al.*, 2016 ▸; Brehm & Diederichs, 2014 ▸; Donatelli & Sethian, 2014 ▸; Liu & Spence, 2014 ▸; Zhou *et al.*, 2013 ▸). *CrystFEL* includes a simplified version of the Brehm–Diederichs algorithm, the details of which have been given previously (White, Mariani *et al.*, 2016 ▸), in the program *ambigator*.

If the space group of the structure under investigation is known in advance, it is easy to know whether an ambiguity is possible or not. A table of symmetry classes (point groups and space groups) is included with *CrystFEL* which highlights the relevant groups. The mathematical basis of an indexing ambiguity, and the manifestation of an indexing ambiguity in the data from an SX experiment, is exactly the same as that of perfect twinning by merohedry (or pseudomerohedry, in the case of approximate overlap of spot positions). Therefore, an indexing ambiguity should be suspected if a ‘twin warning’ is given by the structure-solution software after completing the merging steps described in the next section.

At a minimum, *ambigator* needs the stream file from the previous step (which was called my.stream), the name which should be used for the resulting ‘detwinned’ stream (specified with -o), the true point symmetry of the structure (specified with -y) and either the symmetry operator corresponding to the ambiguity (specified with --operator) or the apparent point symmetry when the ambiguity is in effect (specified with -w):
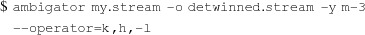
or
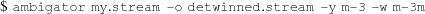
respectively. Typical use will add the further options --fg-graph, --lowres, --highres and -j, which are described below:
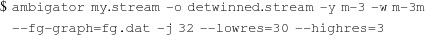
The -j option tells *ambigator* to perform calculations in parallel across multiple CPUs, as described earlier for *indexamajig*. The --fg-graph option tells *ambigator* to write some diagnostic information to a file called fg.dat, which will be described later in this section. The notation used to specify point-group symbols is described in detail in Section 5.2.

The lower and upper resolution limits, given in ångströms using --lowres and --highres, respectively, restrict the range of resolutions used by *ambigator* to calculate correlation coefficients. If the range is too wide, the correlations between data sets can be high even if they are totally un­related, simply because low-resolution reflections are usually strong and high-resolution reflections are usually weak. If the range is too narrow, there will not be enough reflections for an accurate calculation. The best resolution range depends on the data, so it needs to be adjusted based on the results.

An example log output from *ambigator* is shown below:
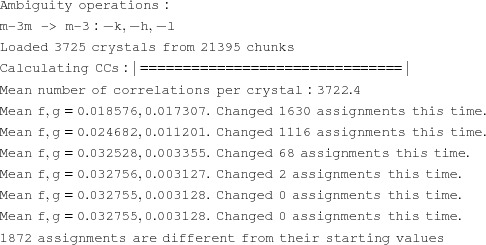
Firstly, the re-indexing operation, calculated from the true and apparent point-group symmetries, is shown.^1^ The most important numbers are the values of the mean correlation coefficients across all crystals in the correct and reversed orientations, called *f* and *g*, respectively. The two values will be similar on the first iteration (here, they are 0.018576 and 0.017307, respectively). If the resolution of the ambiguity is successful, the value of *f* should approximately double while the value of *g* should decrease approximately to zero. Here, the final values are 0.032755 and 0.003128, respectively, indicating a good result. If the final value of *g* is significantly above zero, the resolution range may be too large. If the final value of *f* is not much larger than its starting value, a larger resolution range may be necessary, or the earlier data-processing steps should be revisited.

*Ambigator* will create a new stream (detwinned.stream in the example above) identical to the input stream except that the reflections have been re-indexed and the basis vectors of the reciprocal unit cell have been altered to match. If the ambiguity resolution was successful, this can be merged normally, as described in the next section.

The file fg.dat contains a list of the average correlation coefficients against all other crystals for each crystal, looping round to the first crystal again at the start of the next iteration. This can be used to plot graphs as shown in White, Mariani *et al.* (2016 ▸). For this purpose, a script called *fg-graph* is provided. The script must first be edited to set the filename (usually fg.dat), the number of crystals, the number of iterations (default six) and the minimum and maximum correlation coefficients for the graph axes. Then, it can be run very simply,
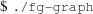
This will produced an image file correlation.png which can be viewed with any image viewer.

*ambigator* attempts to calculate the correlation of the reflection intensities of every crystal with all of the others. In practice, about 1000 correlation coefficients are normally sufficient, depending on the quality and the resolution of the data. If the number of crystals is large, as is the case in the example above, with an average of 3722.4 correlations per crystal, *ambigator* can be told to limit itself to a maximum number of correlation coefficients using the --ncorr option. This will make it finish sooner, usually without affecting the results.

## 5. Merging the measurements   

### 5.1. Checking for detector saturation   

Before merging, it is wise to check for reflections that were too strong to be recorded by the detector. The dynamic range of several current detectors used at XFEL facilities can be as small as a few thousand photons at 8 keV (Carini *et al.*, 2013 ▸), so there are likely to be many overloaded reflections. They can be removed while merging, but the highest reliable pixel value must be known. Unfortunately, the maximum recordable value can vary as the calibration constants for the detector change over a period of days. This is a particular problem for detectors at XFEL facilities.

The *peakogram-stream* script is provided to help to find the saturation value. It simply plots a point for every reflection in a stream, with the horizontal position being the resolution and the vertical position being the maximum intensity of all pixels in the integration region for that reflection. The plot is colour-coded according to the local density of spots. It is simply run on the stream:
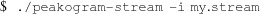



An example graph is shown in Fig. 7 ▸ for a previously published data set from the human serotonin receptor 2B in complex with ergotamine (Liu *et al.*, 2013 ▸), for which the data frames have been made available (White, Barty *et al.*, 2016 ▸). The maximum values for some reflections, particularly at low resolution, have been clipped to lower values. Normally, this would manifest itself as a sharp horizontal line in the graph. However, the image data have been processed to subtract a background value (see Section 2). The background value varies between pixels, but the maximum raw value which can be measured does not. The effective maximum value, which is the maximum raw value minus the background value, therefore also varies between pixels, turning the sharp line into a cloud of points. The maximum reliable value, which is required for the merging step, is simply the highest value before the start of this cloud. In this example, a value of 7000 adu would be appropriate. Note that there are a few outlying values with even higher values of around 14 000 adu in the middle resolution ranges. These come from temporary glitches of the detector and will also be excluded by the cutoff of 7000 adu.

The CSPAD detector (Philipp *et al.*, 2010 ▸) allows pixels to be individually switched between two gain modes, so pixels in one gain mode must have their values multiplied by a certain factor to be consistent with the others. The saturation value is the same in either gain mode, but after performing this multiplication the pixels in high-gain mode would have a much lower saturation value than those in the low-gain mode, for which the pixel values, and therefore the saturation values, have been multiplied up. There are theoretically two possible solutions to this problem. The first is for *CrystFEL* to perform the multiplication, in which case the saturation value would be the same everywhere on the detector, but a gain map would need to be provided to *CrystFEL*. The second option is for the multiplication to be performed by the hit-finding software (see Section 2), in which case *CrystFEL* need not alter the intensity values, but the saturation value will now be different for pixels in one gain mode compared with the other. As a matter of design philosophy, *CrystFEL* adopts the second strategy. To handle the variation in saturation values, a separate HDF5 file can be provided, which matches the layout of the image data and contains the saturation value for each pixel. To assist with creating this map, a script called *gaincal-to-saturation-map* has been provided, but its detailed usage is beyond the scope of this article.

### 5.2. Simple merging using the Monte Carlo method   

The simplest way to merge the intensities is to calculate a average intensity, across the entire data set, for each symmetrically unique reflection. This can be performed using *process_hkl*. For example, to merge my.stream to create merged.hkl using a saturation value of 7000 (see above), merging in point group 4/*mmm*:
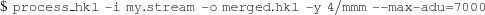



The fact that point group 4/*mmm* is centrosymmetric indicates to *process_hkl* that Friedel pairs are to be merged. To preserve Bijvoet differences in the merged data set, the appropriate symmetry option would be -y 422. The resulting reflection list will contain reflections within the asymmetric unit of reciprocal space for the point group given here. Although perhaps unfamiliar to some users, this way of specifying symmetry is simpler because there are very few point groups compared with space groups. It also avoids any suggestion that the space group is known (or that it needs to be known, or even that guessing it would affect the data processing in any way) at this early stage in the data processing. When specifying the point group for merging, overlines in the point-group symbols are represented by a minus sign, for example point group 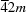 is selected using -y -42m. For trigonal point groups, the type of axes, rhombohedral or hexagonal, must be specified by suffixing with _R or _H, respectively. Unconventional point-group settings can be used, for example point group 6 with the hexagonal axis along *a* can be selected using -y 6_uaa, where the suffix _uaa indicates ‘unique axis *a*’. It should be noted that *CrystFEL* assumes that the unique axis is *c* for all lattice types which have a distinct axis (monoclinic, tetragonal and hexagonal), whereas many other pieces of software prefer the ‘unique axis *b*’ representation for monoclinic structures. To help to avoid confusion, the *CrystFEL* programs will display a warning if a monoclinic point group is used without specifying a unique axis.

### 5.3. Advanced merging using scaling and partiality correction   

In addition to *process_hkl*, the simple merging tool described in the last section, *CrystFEL* includes the more advanced merging program *partialator*. This program can perform more advanced merging methods involving scaling the intensities and correcting for their partialities.

Scaling means to determine scaling factors (linear and Debye–Waller terms) for each crystal which, when the intensities are multiplied by them, bring the individual measurements into as close an agreement as possible. Partiality modelling means accounting for differences in the relative amounts of excitation of the reflections in each diffraction pattern, usually using a geometrical model of the diffraction process (White *et al.*, 2013 ▸; Ginn, Messerschmidt *et al.*, 2015 ▸). Post-refinement means refining the parameters of the model for each crystal such that when the intensities are corrected for their partialities, the measurements agree as closely as possible (Winker *et al.*, 1979 ▸; Rossmann *et al.*, 1979 ▸).

This type of procedure comes at the cost of having to hold the reflection intensities from the entire stream in memory, so that the program can compare them with the intermediate merged results as it iteratively improves the parameters affecting the correction factors. For large unit-cell structures with high-resolution data, this can require large amounts of memory. For 1000 crystals of tetragonal lysozyme (*a* = *b* = 79.2, *c* = 37.9 Å) at a resolution of 3 Å (at the detector edge, not the corner), about half a gigabyte of memory is required. The memory requirements scale linearly with the number of reflections, proportional to each of the unit-cell dimensions and the cube of the maximum resolution (measured as reciprocal distance). Even when modelling partialities and performing post-refinement, *partialator* does not need access to the original image data, only the contents of the stream file. This is the origin of the name ‘post-refinement’: it takes place after all of the other processing is complete (Kabsch, 2010 ▸).

The operation of *partialator* has been described elsewhere (White, 2014 ▸; White, Mariani *et al.*, 2016 ▸). It offers a choice of partiality models, including ‘unity’, which means setting all partialities to 1: in other words, not to model partialities at all and just perform the scaling. Scaling in *partialator* by default determines a linear and Debye–Waller scaling parameter for each crystal to optimize the agreement between it and an intermediate merged data set. The updated parameters are then used to produce the next intermediate merged data set.

The command-line syntax is similar to *process_hkl*:
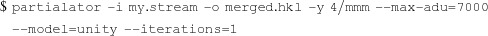
The only changes are the name of the program (*process_hkl* changed to *partialator*) and the addition of --model=unity to specify no partiality modelling and --iterations=1 to specify only one iteration of scaling. The number of iterations refers to the number of ‘macrocycles’ of scaling, post-refinement and outlier rejection. The scaling algorithm itself performs its own iteration cycle within the macrocycles, and therefore only one iteration is sufficient if the ‘unity’ model is used. A higher number of iterations is likely to lead to divergence. If partiality modelling and post-refinement are used, about three iterations should be used.

Although versions of *CrystFEL* going back several years have incorporated partiality models, it is only recently that models have become available that reliably improve experimental data. *CrystFEL* v.0.7.0 includes an implementation of partiality modelling and post-refinement that closely follows that of Ginn, Brewster *et al.* (2015 ▸). This can be used simply by specifying --model=xsphere, as follows:
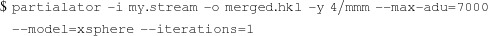
Partiality modelling and post-refinement introduces a lot of complexity into the merging process, and a full guide to the process and discussion of its behaviour would be both beyond the scope of this article and impossible to give at this stage, since the implementation in *CrystFEL* is still considered to be experimental and only works in favourable cases. More detailed discussion will therefore be left for future articles.

**Figure 3:**
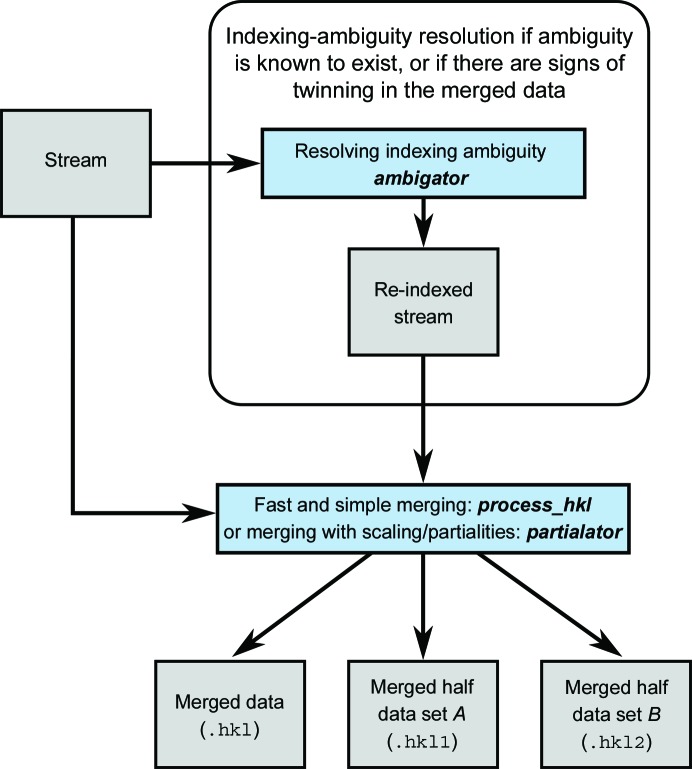
Flow diagram for data merging with *CrystFEL*. See Fig. 2 ▸ for the key to the colours.

The overall flow for merging data, including resolving an indexing ambiguity, is shown in Fig. 3 ▸.

### 5.4. Calculating figures of merit   

**Figure 4:**
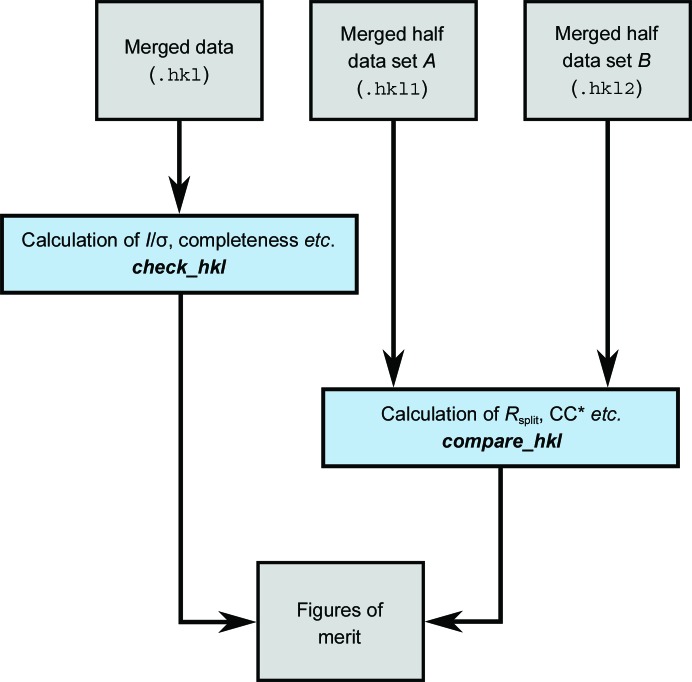
Flow diagram for calculating figures of merit with *CrystFEL*. See Fig. 2 ▸ for the key to the colours.

The workflow for calculating figures of merit in *CrystFEL* is shown in Fig. 4 ▸. There are two programs for calculating figures of merit: *check_hkl* and *compare_hkl*. The former calculates figures of merit relating to just one reflection list, such as completeness and mean *I*/σ(*I*). The latter calculates figures of merit which involve splitting the data set into two halves which are merged separately and then compared, such as *R*_split_ and CC_1/2_.

*check_hkl* is run as follows:
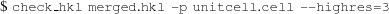
where the --lowres and --highres options can be used to restrict the resolution range which should be considered, since normally the usable data do not extend to the corner of the detector. The program will display several statistics on the terminal, and also create a file shells.dat containing the same statistics divided into resolution bins. The filename of this file can be changed if required, by adding --shell-file=filename, where filename is the desired filename.

*compare_hkl* is run similarly, except that it takes two reflection lists and has an additional option to specify which figure of merit should be calculated:
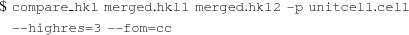
Possible figures of merit include *R*_split_, CC, CC* and *R*_ano_; a full list can be found in the manual page (man compare_hkl). It also writes shells.dat, and the filename can be altered as with *check_hkl*. In the example above, the correlation coefficient is being calculated between two half data sets; therefore the resulting values are CC_1/2_. This, or the closely related CC* (Karplus & Diederichs, 2012 ▸), are the preferred figures of merit (over *R*_split_) for estimating the resolution limit of useful data (Karplus & Diederichs, 2015 ▸).

The two merged half data sets, here called merged.hkl1 and merged.hkl2, will automatically be created by *partialator*. To create them using *process_hkl* is more difficult, and requires that the program be run two more times in addition to the main merge, once using the even-numbered images and once using the odd-numbered images,
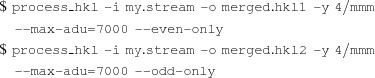
The designations ‘odd’ and ‘even’ refer to the order that the crystals appear in the stream, which is effectively random owing to the variable amount of time taken to process each frame in parallel by *indexamajig*.

The symmetry used for merging is written into the merged reflection files, and therefore does not need to be given to *check_hkl* and *compare_hkl* as was the case in earlier versions of *CrystFEL*.

### 5.5. Exporting to structure-solution packages   

**Figure 5:**
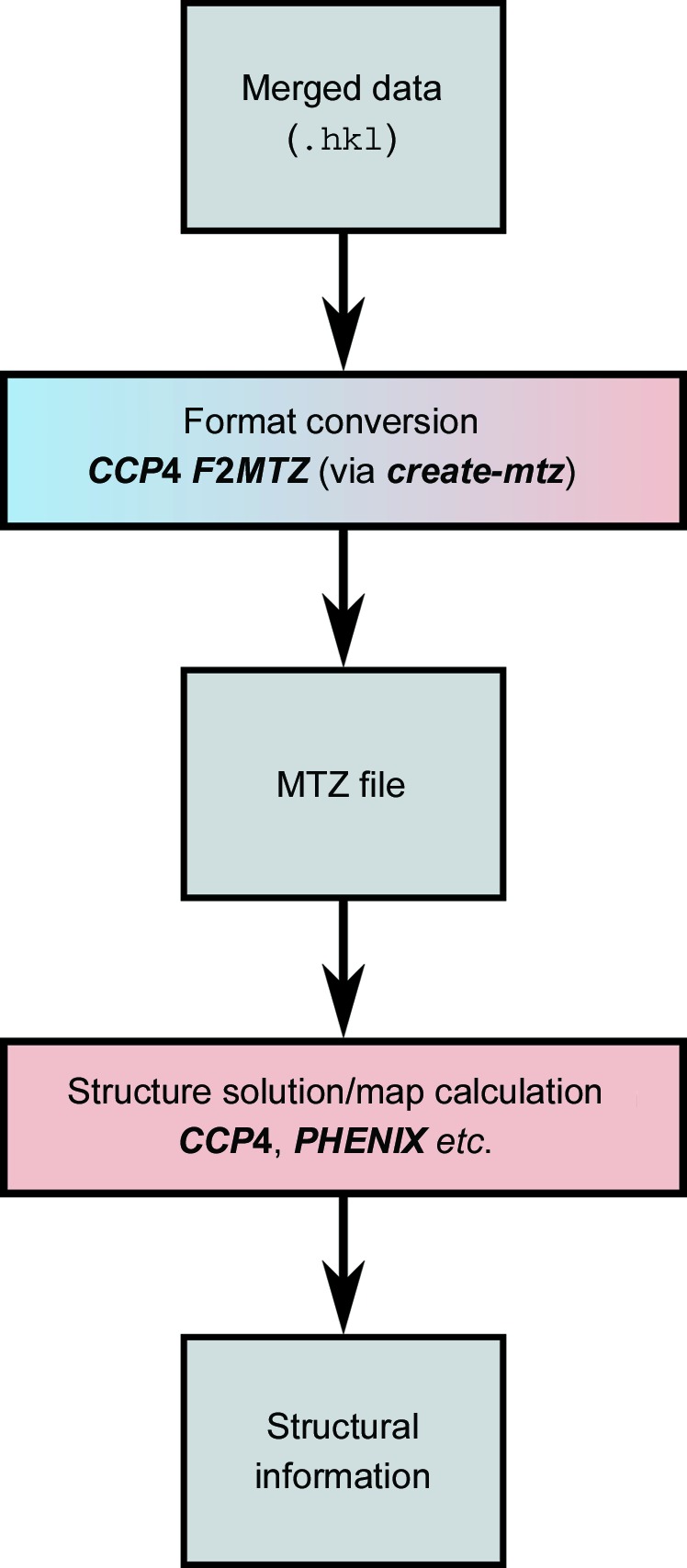
Flow diagram of data export from *CrystFEL*. See Fig. 2 ▸ for the key to the colours. The gradient-shaded blue/red box indicates an external program (*F*2*MTZ* from *CCP*4) being driven by a *CrystFEL* script (*create-mtz*).

**Figure 6:**
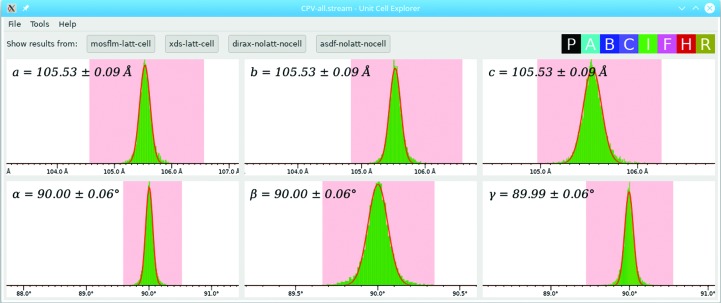
Screenshot of *cell_explorer*, showing clear peaks for each of the six unit-cell parameters, each of which has been selected and fitted.

**Figure 7:**
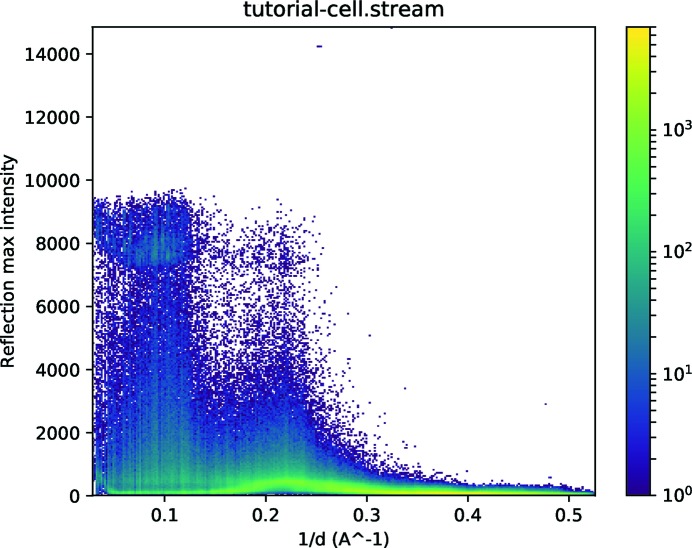
Intensity plot created by the *peakogram-stream* script, showing a cloud of points owing to saturation at low resolution between 7000 and 10 000 intensity units.

The merged reflection files in *CrystFEL* are plain text and can easily be imported into most structure-solution packages. Nevertheless, template scripts are provided to help. The scripts are *create-mtz* and *create-xscale* to create MTZ files and *XSCALE* files, respectively. The workflow for using *create-mtz* is shown in Fig. 5 ▸. *create-mtz* is a thin wrapper around the *CCP*4 import program *F*2*MTZ*, and *create-xscale* is a simple Perl script because *XSCALE* files are also plain text. Both scripts must be carefully edited before use to set the unit-cell parameters, data-set name and other values which will be written into the headers of the resulting file.

## 6. Additional features   

At this stage, the processing of the data is essentially complete. However, there are many other features in *CrystFEL* which become useful for certain usage cases. The next few sections contain brief introductions to some of the most important ones.

### 6.1. Custom data-set splitting   

Several types of experiment, such as time-resolved experiments or multi-data-set anomalous phasing experiments (SIR or MAD), involve multiple data sets which have small differences between them. For these types of experiment, it is important to ensure that all data sets are processed identically. To support this, *partialator* offers the possibility of merging all of the data together in one combined task and then separating the data sets just before creating the final merged output. In this way, the separate data sets are certain to be on the same scale, and concerns about the uniqueness of the solution when using post-refinement are greatly reduced.

To use this feature, a separate file must be provided to *partialator* using the option --custom-split=filename. The file consists of one line per frame, each line consisting of the filename, frame identifier if applicable (see Section 2) and an arbitrary data-set identifier. The identifier can be any text string, for example ‘native’, ‘derivative’, ‘dark’, ‘light_1ns’ and so on. For each unique data-set identifier, three extra files will be written. In the example above, where the main merged output was merged.hkl, for a data-set identifier ‘native’ the extra files would be called merged-native.hkl, merged-native.hkl1 and merged-native.hkl2. These files contain the merged data for the data set (.hkl) and two half-data-set merges (.hkl1 and .hkl2) which can be used to calculate figures of merit for that data set alone.

### 6.2. Searching for ‘mini rotation series’   

Whereas an XFEL pulse will usually destroy the crystal, in synchrotron serial crystallography experiments there is a chance that the same crystal might appear in two or more successive frames. This knowledge can be useful, for example to treat the accidental successive exposures as a rotation series and process them with rotation-processing software, leading to a hybrid approach similar to that described by Gati *et al.* (2014 ▸). The information could also be used to avoid subsequent exposures in case they have suffered unacceptable levels of radiation damage. To this end, the *whirligig* program in *CrystFEL* can scan through a stream file and compare the orientations of crystals in subsequent frames. The program takes into account that there might be multiple crystals per frame, that the frames might not appear in the stream in the same order that they were mentioned in the input list (see Section 2) and also that the indexing of the series of crystals may not be consistent. It is very easy to run:
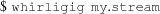



The only parameter that may need to be adjusted is the ‘window size’, which determines how widely separated two successive frames can be in the stream before the program abandons hope that they will appear. The program will report if this is necessary.

For each series of crystals, a short text file will be written which contains the filenames, event descriptors and crystal numbers which make up the series. The example output below could arise from a series of three patterns, their filenames being frame_00897.cbf, frame_00898.cbf and frame_00899.cbf:
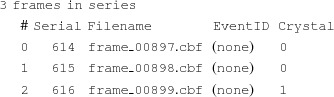
Here, the last frame in fact contains two crystals, the second of which was apparently close in orientation to the previous crystal. The crystal indices start from zero, and the text ‘(none)’ would have been replaced by an event descriptor if the input files contained more than one frame each.

### 6.3. Running *CrystFEL* on a cluster system   

Many of the *CrystFEL* programs support the use of the option -j to instruct them to perform multiple parts of their work in parallel. However, this parallelism only uses multiple CPUs within one computer. This is usually sufficient, except for the indexing and integration step. This is usually the most time-consuming part of processing an SX data set, firstly because of the amount of work involved (potentially including several attempts at indexing each pattern) and secondly because it involves reading the image data for every frame, creating a large input/output load. This step can be made much faster by parallelizing not only within one computer, but across many computers in a cluster, as are available at most X-ray light-source facilities.

Splitting the indexing and integration task up across computers can be performed by creating many small *indexamajig* subtasks with separate input file lists (see Section 2), each containing about 1000 frames from the full data set. The tasks are submitted to a batch-queuing system which takes care of scheduling and running them on the cluster. Each subtask produces its own stream, and the streams can be combined by simple concatenation. For example, if the substreams have names block00.stream, block01.stream, block02.stream and so on, this could be performed with
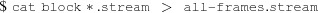
There is no conceptual difference between the substreams, the combined stream and the stream obtained by a single large run of *indexamajig*. The combined stream can be used exactly as described above for merging the data, or the substreams can be used to examine the indexing results for a subset of the complete data.

As is perhaps already apparent, speeding up the processing in this way comes at the cost of convenience. To assist, two scripts are provided with *CrystFEL*, which should be considered as templates and customised to the exact task. The two scripts are called *turbo-index-slurm* and *turbo-index-lsf*, and are for systems using the SLURM (https://slurm.schedmd.com/) and Platform LSF batch-queue systems, respectively. A full tutorial of how to modify and run these scripts is beyond the scope of this article, but they must be modified to set parameters including the number of frames per subtask, the *indexamajig* command-line parameters and any environment setup needed to run *CrystFEL* on the worker nodes, as well any parameters needed by the batch system such as an e-mail address for notifications, job priority and expected running time.

## 7. Conclusion   

This step-by-step guide has covered the main processing pipeline for serial crystallography data using *CrystFEL*. Most, but not all, of the core programs in *CrystFEL* have been described. Those not covered are those for simulating diffraction data, which can be used for testing new types of analysis or determining expected signal levels for experiments. The programs for simulation are *pattern_sim* and *partial_sim*, which have both been described previously (White *et al.*, 2013 ▸). There is also *render_hkl*, which can plot plane sections through reciprocal space, showing the intensities of reflections using a colour scale. The remaining program is *get_hkl*, which can be used for manipulating reflection lists, for example symmetry expansion, adding noise, applying a resolution cutoff or re-indexing a reflection list. These functions are also most commonly used when simulating data.

The library of scripts provided with *CrystFEL* is much larger than those described here. A list is given below, with brief descriptions of the most important of the scripts which have not so far been mentioned. Although some of these refer to features which have not been described above, they are mentioned here to make the reader aware of their existence.

*ave-resolution*: plot a histogram of the estimated resolution of the crystals in a stream and show the average and maximum values.
*eiger-badmap*: create a bad pixel mask for a Dectris EIGER detector by finding very bright pixels in a single data file.
*move-entire-detector*: apply an overall shift to all panels in a detector geometry file at once.
*mtz*2*hkl*: create a *CrystFEL* reflection file from an MTZ file; the opposite of *create-mtz* described above.
*stream_grep*: search a stream for chunks which match certain criteria and write a new, filtered stream.
*sum-peaks*: create an image (in HDF5 format) where a single pixel is plotted for each of the peaks in a stream. This image can then be used to adjust the detector geometry.
*truncate-stream*: extract a subset of chunks or crystals from a stream with a specified start point and length.

Also not mentioned is the *CrystFEL* shared library, called libcrystfel, which can be used to write separate programs in C or C++ which make use of the *CrystFEL* data structures. libcrystfel is not intended as a replacement for more complete crystallography libraries such as *cctbx* (Grosse-Kunstleve *et al.*, 2002 ▸) or the CCP4 libraries (Winn *et al.*, 2011 ▸), but rather as a way of supporting access to high-level functions such as the indexing system, for example when interfacing with online data-analysis frameworks at light-source facilities.

As with all software, things change quickly as new features are added. The *CrystFEL* website features a tutorial which is kept up to date with the latest changes. The website also features a list of changes between versions, which can be consulted to quickly see where differences should be expected. Finally, ‘release notes’ are published for each new version, which contains details of the larger changes and new features.

*Note added in proof*: A new version of *CrystFEL* (0.8.0) was released shortly before this article went to press. See the *CrystFEL* website (https://www.desy.de/~twhite/crystfel) for more details.

## Supplementary Material

Worked example for human serotonin receptor 2B data.. DOI: 10.1107/S205979831801238X/ba5291sup1.pdf

Geometry file for human seotonin receptor 2B data, updated for CrystFEL v.0.7.0.. DOI: 10.1107/S205979831801238X/ba5291sup2.txt

## Footnotes

1 As a convenience, the ‘twin law’ relating any two point groups can be calculated by invoking *ambigator* with only the apparent and true symmetries; for example,  ambigator -y m-3 -w m-3m.
