# Supplementary material for: Processing serial crystallography data with CrystFEL: a step-by-step guide
Source: Acta Crystallogr D Struct Biol. 2019 Jan 31;75(Pt 2):219–33. doi: 10.1107/S205979831801238X (PMC6400257; doi:10.1107/S205979831801238X)
Supplement: Supplementary file 1 [file d-75-00219-sup1.pdf]

STRUCTURAL BIOLOGY

Volume 75 (2019)

Supporting information for article:

Processing serial crystallography data with *CrystFEL*: a step-by-step guide

Thomas A. White

Worked example for 5-HT_{2B} receptor dataset

This worked example includes the complete processing, including the exact sequence of commands and expected outcomes, for some data freely available on the Coherent X-ray Imaging Data Bank (CXIDB).

It will be assumed that CrystFEL version 0.7.0 has been installed. Commands to be typed are shown in bold following the prompt symbol "$". Newlines in commands are for clarity only, and should not be typed. "[...]" indicates that some of the output from the command has been omitted for clarity.

The frames were acquired at LCLS from crystals of the serotonin 5-HT_{2B} receptor bound to ergotamine. The complete data consists of over 150,000 frames (after filtering out blank frames), but only a small sample of 5775 frames will be used here. The processing here is not claimed to be optimal, but serves to illustrate the use of the software.

More information about the data can be found in the following articles:

- W. Liu, D. Wacker, C. Gati, G. W. Han et al. "Serial Femtosecond Crystallography of G Protein-Coupled Receptors". Science 342 (2013) p1522. doi:10.1126/science.1244142.
- T. A. White, A. Barty, W. Liu, A. Ishchenko et al. "Serial femtosecond crystallography datasets from G protein-coupled receptors". Scientific Data 3:160057. 10.1038/sdata.2016.57.
- Link to the data on the CXIDB: http://www.cxidb.org/id-21.html

Check version of CrystFEL:

```
$ indexamajig --version
CrystFEL: 0.7.0
License GPLv3+: GNU GPL version 3 or later <http://gnu.org/licenses/gpl.html>.
This is free software: you are free to change and redistribute it.
There is NO WARRANTY, to the extent permitted by law.

Written by Thomas White and others.
```

Download and unpack the data:

```
$ wget http://cxidb.org/data/21/cxidb-21-run0130.tar
[...]
$ tar -xf cxidb-21-run0130.tar
```

Download initial geometry file. This geometry file is the same as the one available from the CXIDB website for this dataset, but has been updated for compatibility with CrystFEL 0.7.0. It is also available as supporting information to the article.

```
$ wget https://www.desy.de/~twhite/crystfel/5HT2B-Liu-2013.geom
```

Create the list of files to process and count the number of files:

```
$ find cxidb-21-run013* -name 'LCLS*.h5' -print > files.lst
$ wc -l files.lst
5775 files.lst
```

There are 5775 files to process. In this case, each file corresponds to a single data frame, but this need not be the case (see section 2 of the article).

Create an initial stream with peak detection, no indexing nor integration:

```
$ indexamajig -i files.lst -g 5HT2B-Liu-2013.geom --peaks=zaef --threshold=300
       --min-gradient=500000 --min-snr=5 --int-radius=3,4,5 --indexing=none
       -o tutorial.stream -j 4
Indexing/integration disabled.
   0 indexable out of   67 processed ( 0.0%),    0 crystals so far.   67 images processed since the last message.
   0 indexable out of  143 processed ( 0.0%),    0 crystals so far.   76 images processed since the last message.
   0 indexable out of  211 processed ( 0.0%),    0 crystals so far.   68 images processed since the last message.
   0 indexable out of  277 processed ( 0.0%),    0 crystals so far.   66 images processed since the last message.
[...]
   0 indexable out of 5698 processed ( 0.0%),    0 crystals so far.   63 images processed since the last message.
   0 indexable out of 5767 processed ( 0.0%),    0 crystals so far.   69 images processed since the last message.
Waiting for the last patterns to be processed...
Final: 5775 images processed, 0 had crystals (0.0%), 0 crystals overall.
```

Alternatively, using the peak location information stored in the data files (program output should be similar to the above):

```
$ indexamajig -i files.lst -g 5HT2B-Liu-2013.geom --peaks=hdf5 --indexing=none
       -o tutorial.stream -j 4
[...]
```

Check the spot finding:

```
$ cp ~/crystfel/scripts/check-peak-detection .
$ chmod +x check-peak-detection
$ ./check-peak-detection tutorial.stream -g 5HT2B-Liu-2013.geom --int-boost=5
Extra arguments for hdfsee: -g 5HT2B-Liu-2013.geom --int-boost=5
Viewing cxidb-21-run0131/data1/LCLS_2013_Mar23_r0131_003712_4e09.h5
Viewing cxidb-21-run0131/data1/LCLS_2013_Mar23_r0131_003844_d09b.h5
Viewing cxidb-21-run0131/data1/LCLS_2013_Mar23_r0131_003834_c195.h5
```

Close the hdfsee window each time to view the next frame. Press Ctrl+C in the terminal to stop.

Indexing and integration using spot location information in data files, no prior information, no unit cell restrictions and automatic selection of indexing methods:

```
$ indexamajig -i files.lst -g 5HT2B-Liu-2013.geom --peaks=hdf5 --int-radius=3,4,5 -o tutorial.stream -j 4
No indexing methods specified.  I will try to automatically detect the available methods.
To disable auto-detection of indexing methods, specify which methods to use with --indexing=<methods>.
Use --indexing=none to disable indexing and integration.
No reference unit cell provided.
WARNING: Forcing --no-check-cell because reference unit cell parameters were not given.
WARNING: Forcing all indexing methods to use "-nocell", because reference cell parameters were not given.
List of indexing methods:
   0: mosflm-nolatt-nocell      (mosflm - no prior information)
   1: dirax-nolatt-nocell       (dirax - no prior information)
   2: asdf-nolatt-nocell        (asdf - no prior information)
   3: xds-nolatt-nocell         (xds - no prior information
Indexing parameters:
                  Check unit cell parameters: off
                        Check peak alignment: on
                   Refine indexing solutions: on
 Multi-lattice indexing ("delete and retry"): off
                              Retry indexing: on
   2 indexable out of    4 processed (50.0%),    2 crystals so far.    4 images processed since the last message.
   3 indexable out of    6 processed (50.0%),    3 crystals so far.    2 images processed since the last message.
[...]
5286 indexable out of 5763 processed (91.7%), 5286 crystals so far.    6 images processed since the last message.
5289 indexable out of 5766 processed (91.7%), 5289 crystals so far.    3 images processed since the last message.
Waiting for the last patterns to be processed...
WARNING: 1 implausibly negative reflection in cxidb-21-run0139/data1/LCLS_2013_Mar23_r0139_021534_bc2b.h5 (none)
Final: 5775 images processed, 5298 had crystals (91.7%), 5298 crystals overall.
```

Warnings of "implausibly negative reflections" can be ignored at this stage.

Determination of unit cell parameters:

```
$ cell_explorer tutorial.stream
Loaded 5298 cells from 5775 chunks
Selected 5298 of 5298 cells
```

Select dominant peaks for each parameter, fit and create unit cell file under "5HT2B.cell". Edit this file with a text editor to fix small errors in angles, in this case, set them all to 90°:

```
$ vim 5HT2B.cell
$ cat 5HT2B.cell
CrystFEL unit cell file version 1.0

lattice_type = orthorhombic
centering = C
a = 61.72 A
b = 122.98 A
c = 168.34 A
al = 90.00 deg
be = 90.00 deg
ga = 90.00 deg
```

Index using known unit cell:

```
$ indexamajig -i files.lst -g 5HT2B-Liu-2013.geom --peaks=hdf5
       --int-radius=3,4,5 -o tutorial.stream -p 5HT2B.cell -j 4
No indexing methods specified.  I will try to automatically detect the available methods.
To disable auto-detection of indexing methods, specify which methods to use with --indexing=<methods>.
Use --indexing=none to disable indexing and integration.
This is what I understood your unit cell to be:
orthorhombic C, unique axis ?, right handed.
a      b      c            alpha   beta  gamma
 61.72 122.98 168.34 A     90.00  90.00  90.00 deg
a =  6.172e-09  0.000e+00  0.000e+00 m
b =  7.530e-25  1.230e-08  0.000e+00 m
c =  1.031e-24  1.031e-24  1.683e-08 m
a* =  1.620e+08 -9.921e-09 -9.921e-09 m^-1 (modulus  1.620e+08 m^-1)
b* =  0.000e+00  8.131e+07 -4.979e-09 m^-1 (modulus  8.131e+07 m^-1)
c* =  0.000e+00  0.000e+00  5.940e+07 m^-1 (modulus  5.940e+07 m^-1)
alpha* =  90.00 deg, beta* =  90.00 deg, gamma* =  90.00 deg
Cell representation is crystallographic, direct space.
List of indexing methods:
   0: mosflm-nolatt-cell        (mosflm using cell parameters as prior information)
   1: mosflm-latt-nocell        (mosflm using Bravais lattice type as prior information)
   2: dirax-nolatt-nocell       (dirax - no prior information)
   3: asdf-nolatt-cell          (asdf using cell parameters as prior information)
   4: xds-latt-cell             (xds using cell parameters and Bravais lattice type as prior information)
Indexing parameters:
                  Check unit cell parameters: on (axis combinations)
                        Check peak alignment: on
                   Refine indexing solutions: on
 Multi-lattice indexing ("delete and retry"): off
                              Retry indexing: on
   0 indexable out of    2 processed ( 0.0%),    0 crystals so far.    2 images processed since the last message.
   1 indexable out of    7 processed (14.3%),    1 crystals so far.    5 images processed since the last message.
   3 indexable out of   13 processed (23.1%),    3 crystals so far.    6 images processed since the last message.
[...]
3729 indexable out of 5766 processed (64.7%), 3729 crystals so far.    3 images processed since the last message.
3731 indexable out of 5769 processed (64.7%), 3731 crystals so far.    3 images processed since the last message.
Waiting for the last patterns to be processed...
Final: 5775 images processed, 3733 had crystals (64.6%), 3733 crystals overall.
```

Check beam centre position and update geometry file:

```
$ cp ~/crystfel/scripts/detector-shift .
$ chmod +x detector-shift
$ ./detector-shift tutorial.stream 5HT2B-Liu-2013.geom
Mean shifts: dx = -0.12 mm,  dy = -0.22 mm
Applying corrections to 5HT2B-Liu-2013.geom, output filename 5HT2B-Liu-2013-predrefine.geom
Using default resolution (9097.525473 px/m) for panel q0a0
Using default resolution (9097.525473 px/m) for panel q0a0
[...]
```

Observe that cloud of points is not centered on the origin, and that an updated geometry file has been written as 5HT2B-Liu-2013-predrefine.geom. Repeat indexing and integration using improved geometry file:

```
$ indexamajig -i files.lst -g 5HT2B-Liu-2013-predrefine.geom --peaks=hdf5
       --int-radius=3,4,5 -o tutorial.stream -p 5HT2B.cell -j 4
No indexing methods specified.  I will try to automatically detect the available methods.
To disable auto-detection of indexing methods, specify which methods to use with --indexing=<methods>.
Use --indexing=none to disable indexing and integration.
This is what I understood your unit cell to be:
[...]
4112 indexable out of 5764 processed (71.3%), 4112 crystals so far.    7 images processed since the last message.
4115 indexable out of 5771 processed (71.3%), 4115 crystals so far.    7 images processed since the last message.
Waiting for the last patterns to be processed...
Final: 5775 images processed, 4117 had crystals (71.3%), 4117 crystals overall.
```

Check predicted spot positions:

```
$ cp ~/crystfel/scripts/check-near-bragg .
$ chmod +x check-near-bragg
$ ./check-near-bragg tutorial.stream -g 5HT2B-Liu-2013.geom
```

Check saturation:

```
$ cp ~/crystfel/scripts/peakogram-stream .
$ chmod +x peakogram-stream
$ ./peakogram-stream -i tutorial.stream
1712651 predicted reflections found
```

Simple merging:

```
$ process_hkl -i tutorial.stream -o tutorial.hkl -y 222 --max-adu=7000
5775 images processed, 4117 crystals, 4117 crystals used.
$ process_hkl -i tutorial.stream -o tutorial.hkl1 -y 222 --max-adu=7000 --even-only
5775 images processed, 4117 crystals, 2058 crystals used.
$ process_hkl -i tutorial.stream -o tutorial.hkl2 -y 222 --max-adu=7000 --odd-only
5775 images processed, 4117 crystals, 2059 crystals used.
```

Advanced merging:

```
$ partialator -i tutorial.stream -o tutorial.hkl -y 222 --iterations=1 --model=unity --max-adu=7000 -j 4
Setting --no-pr because we are not modelling partialities (--model=unity).
5775 images loaded, 4117 crystals.
Initial partiality calculation...
Initial scaling...
Scaling: |==================================================|
Log residual went from 1.911395e+05 to 1.305348e+05, 4117 crystals
Mean B = -3.544361e-20
Scaling: |==================================================|
Log residual went from 1.405601e+05 to 1.250560e+05, 4117 crystals
Mean B = -1.304877e-19
Scaling: |==================================================|
Log residual went from 1.390499e+05 to 1.250792e+05, 4117 crystals
Mean B = -2.262129e-19
21 bad crystals:
  4096 OK
  21 B too big
Residuals:  linear          linear free     log             log free
            3.319215e+03    3.437622e+03    4.842531e+05    2.432335e+04
Scaling and refinement cycle 1 of 1
Scaling: |==================================================|
Log residual went from 1.388096e+05 to 1.250602e+05, 4096 crystals
Mean B = -3.214438e-19
Scaling: |==================================================|
Log residual went from 1.388158e+05 to 1.251237e+05, 4096 crystals
Mean B = -4.162659e-19
41 bad crystals:
4076 OK
41 B too big
Residuals:  linear          linear free     log             log free
            2.544917e+03    3.311812e+03    4.804008e+05    2.391690e+04
Final merge...
Scaling: |==================================================|
Log residual went from 1.385992e+05 to 1.249499e+05, 4076 crystals
Mean B = -5.108462e-19
Scaling: |==================================================|
Log residual went from 1.386145e+05 to 1.249749e+05, 4076 crystals
Mean B = -6.053857e-19
Residuals:  linear          linear free     log             log free
            2.470401e+03    3.472257e+03    4.796287e+05    2.389876e+04
Writing overall results to tutorial.hkl
Writing two-way split to tutorial.hkl1 and tutorial.hkl2
```

Figures of merit based on merged data, in 20 resolution bins up to 3 Å resolution:

```
$ check_hkl tutorial.hkl -y 222 -p 5HT2B.cell --highres=3 --nshells=20
Discarded 0 reflections (out of 81598) with I/sigma(I) < -inf
56798 reflections rejected because they were outside the resolution range.
1/d goes from 0.287938 to 3.333322 nm^-1
overall <snr> = 2.245886
929840 measurements in total.
24800 reflections in total.
Resolution shell information written to shells.dat.
$ cat shells.dat
```

Center 1/nm	# refs	Possible	Compl	Meas	Red	SNR	Std dev	Mean	d(A)	Min 1/nm	Max 1/nm
0.760	1265	1265	100.00	123441	97.6	8.17	16157.91	18752.09	13.16	0.287	1.233
1.392	1235	1235	100.00	58917	47.7	4.89	7533.70	6459.03	7.19	1.233	1.550
1.662	1222	1222	100.00	66675	54.6	4.32	3503.46	3149.84	6.02	1.550	1.773
1.862	1258	1258	100.00	64155	51.0	4.21	4467.30	3830.91	5.37	1.773	1.951
2.026	1235	1235	100.00	52497	42.5	4.12	4594.57	4634.09	4.94	1.951	2.101
2.167	1245	1245	100.00	51403	41.3	3.50	3965.85	3911.61	4.61	2.101	2.233
2.291	1231	1231	100.00	50703	41.2	2.95	2626.06	2536.41	4.36	2.233	2.350
2.403	1247	1247	100.00	42122	33.8	2.39	2023.33	1766.73	4.16	2.350	2.457
2.506	1261	1261	100.00	37304	29.6	1.63	1209.15	890.08	3.99	2.457	2.555
2.601	1237	1237	100.00	39941	32.3	1.62	836.49	674.20	3.85	2.555	2.646
2.689	1213	1213	100.00	39545	32.6	1.46	615.57	461.14	3.72	2.646	2.732
2.772	1259	1259	100.00	39715	31.5	1.09	410.40	267.60	3.61	2.732	2.812
2.850	1214	1214	100.00	37338	30.8	0.96	292.98	186.58	3.51	2.812	2.888
2.924	1251	1251	100.00	37574	30.0	0.87	249.42	139.20	3.42	2.888	2.960
2.994	1238	1238	100.00	33325	26.9	0.69	181.24	91.05	3.34	2.960	3.029
3.062	1245	1245	100.00	31016	24.9	0.67	169.52	80.60	3.27	3.029	3.095
3.126	1231	1231	100.00	31192	25.3	0.39	105.36	37.08	3.20	3.095	3.158
3.188	1219	1219	100.00	31306	25.7	0.37	91.92	29.47	3.14	3.158	3.218
3.248	1289	1289	100.00	32200	25.0	0.27	81.81	19.30	3.08	3.218	3.277
3.305	1205	1205	100.00	29471	24.5	0.23	76.59	14.28	3.03	3.277	3.333

Calculation of CC$_{1/2}$ based on half-dataset correlation, again in 20 resolution bins up to 3 Å resolution:

```
$ compare_hkl tutorial.hkl1 tutorial.hkl2 -y 222 -p 5HT2B.cell --fom=cc --highres=3 --nshells=20
tutorial.hkl1: 73569 reflections, resolution range 34.73 to 1.90 Angstroms (0.28794 to 5.24997 nm^-1).
tutorial.hkl2: 72998 reflections, resolution range 34.73 to 1.90 Angstroms (0.28794 to 5.25612 nm^-1).
42772 reflection pairs rejected because either or both versions were outside the resolution range.
24735 reflection pairs accepted.
Accepted resolution range: 0.287938 to 3.333322 nm^-1 (34.73 to 3.00 Angstroms).
Fixed resolution range: 0.287938 to 3.333333 nm^-1 (34.73 to 3.00 Angstroms).
Relative scale factor = 1.018431, relative B factor = -0.714309 A^2
A scale factor greater than 1 means that the second reflection list is weaker than the first.
A positive relative B factor means that the second reflection list falls off with resolution more quickly than the first.
Overall CC = 0.9589985
$ cat shells.dat
```

1/d centre	CC	nref	d / A	Min 1/nm	Max 1/nm
0.760	0.9485312	1265	13.15	0.288	1.233
1.392	0.9212586	1232	7.19	1.233	1.550
1.662	0.8890394	1223	6.02	1.550	1.773
1.862	0.9075102	1256	5.37	1.773	1.951
2.026	0.8609918	1234	4.94	1.951	2.101
2.167	0.8596543	1245	4.61	2.101	2.233
2.291	0.8006276	1231	4.36	2.233	2.350
2.403	0.7737574	1245	4.16	2.350	2.457
2.506	0.6272112	1253	3.99	2.457	2.555
2.601	0.5855905	1233	3.85	2.555	2.646
2.689	0.5973667	1208	3.72	2.646	2.732
2.772	0.4832503	1256	3.61	2.732	2.812
2.850	0.3774442	1211	3.51	2.812	2.888
2.924	0.3159704	1251	3.42	2.888	2.960
2.994	0.2466243	1228	3.34	2.960	3.029
3.062	0.3032666	1240	3.27	3.029	3.095
3.126	0.0963166	1224	3.20	3.095	3.158
3.188	0.0173747	1215	3.14	3.158	3.218
3.248	0.0780959	1285	3.08	3.218	3.277
3.305	0.0524823	1200	3.03	3.277	3.333
